# The protective effects of butorphanol tartrate against homocysteine-induced blood-brain barrier dysfunction

**DOI:** 10.1080/21655979.2022.2037953

**Published:** 2022-03-04

**Authors:** Sufeng Shen, Jiandong Wang, Qingyuan Zhao, Qiangfu Hu

**Affiliations:** Department of Anesthesiology, Fifth Affiliated Hospital of Zhengzhou University, Zhengzhou City, Henan Province, China

**Keywords:** Stroke, blood-brain barrier, butorphanol tartrate, claudin-5, KLF5

## Abstract

A high concentration of homocysteine (Hcy) has been recently reported to be closely associated with the development of stroke, which is related to the Hcy-induced blood-brain barrier (BBB) dysfunction. Butorphanol tartrate is a promising analgesic agent that targets the opiate receptor and shows promising protective effects on ischemia/reperfusion injury. The present research proposes to investigate the protective effect of butorphanol tartrate on Hcy-induced BBB disruption to explore the potential application of butorphanol tartrate in treating Hcy-induced stroke. Hcy was utilized to establish both an *in vivo* animal model and *in vitro* human brain vascular endothelial cells (HBVECs) injury model. We found that the increased diffusion of sodium fluorescein and Evan’s blue, declined expression of Claudin-5, and increased production of interleukin- 6 (IL-6) and tumor necrosis factor-α (TNF-α) were observed in Hcy-treated mice, which were all significantly reversed by butorphanol tartrate. In Hcy-stimulated HBVECs, increased endothelial permeability and reduced expression levels of Claudin-5 and Krüppel-like factor 5 (KLF5) were observed, all of which were dramatically rescued by 2 and 5 µM butorphanol tartrate. Lastly, the protective function of butorphanol tartrate in Hcy-stimulated HBVECs was dramatically abolished by the knockdown of KLF5. Collectively, butorphanol tartrate showed protective effects on Hcy-induced BBB disruption by upregulating the KLF5/Claudin-5 axis.

## Graphical Abstract

**Figure:**
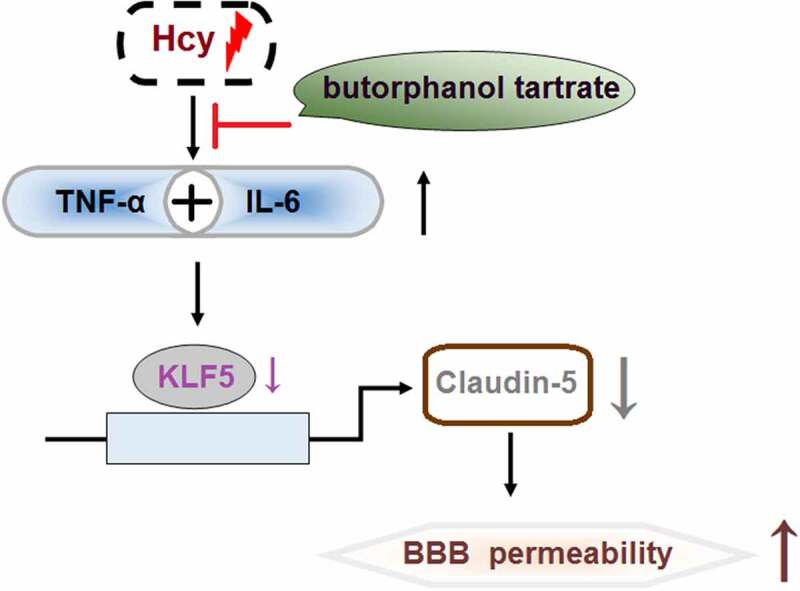

## Introduction

Recently, homocysteine (Hcy) has been gradually recognized as an important inducer for the development of multiple types of cardiovascular diseases, such as atherosclerosis, stroke, and venous thrombosis [1]. It is reported that Hcy impacts metabolic oxidation and induces the accumulation of local platelets by facilitating the proliferation of vascular smooth muscle cells and inducing damage to vascular endothelial cells [2]. The expression level of the synthetase of nitric oxide (NO) is reported to be repressed by Hcy to reduce the production of NO, which triggers the injury on endothelial cells and impacts vasodilatation. In addition, thromboxane (TXA2) is found to be upregulated by Hcy and subsequently, vasoconstriction is induced and the platelets activated, contributing to the imbalance of the coagulation and fibrinolysis mechanisms [3]. Chronic inflammation on artery walls can be induced by Hcy to facilitate the progression of atherosclerosis, resulting in the development of stroke [4]. It is reported that each 5 μmol/L increase in Hcy increases the incidence of ischemic stroke by 15% [5]. Previous researches have already implied that the development of stoke can be induced by the disrupted blood-brain barrier (BBB) triggered by Hcy [6]. The BBB is mainly composed of vascular endothelial cells, tight junctions (TJs), and perithelial cells, all of which are critical for the controlling materials entering brain tissues from the circulation [7]. Recently, it has become widely accepted that the dysfunction of the BBB is a vital inducer for stroke [8,9]. Hwayong Lee reported that amorphous fibrosis was observed in the basal membranes of the hippocampal CA1 regions of the brains in rabbits treated with Hcy, accompanied by the irregular thickening of the basal membranes of the cerebral microvessels, separation of the peripheral capillaries, and degeneration of perithelial cells [10]. Beard further verified that disruption of the BBB induced by Hcy was closely associated with the dysfunction of tight junctions [11]. Recently, downregulated claudin-5 is found to be an important factor involved in the regulatory function of Hcy on tight junctions [12]. Therefore, it is critical to protect BBB integrity by maintaining the function of tight junctions for the treatment of Hcy-induced stroke. Kruppel-like factor 5 (KLF5) is a transcriptional factor involved in regulating the expression of multiple genes. Recently, it is reported that KLF5 is a positive factor for the maintenance of BBB integrity and function of tight junctions [13].

Butorphanol tartrate is a mixed agonist-antagonist of opiate receptors, activating the κ receptor and inactivating the μ receptor. The analgesic effect of butorphanol tartrate is 4-5-fold higher than that of morphine [14]. Butorphanol tartrate has achieved promising therapeutic effects during its clinical application [15]. Recently, it is reported to show significant therapeutic effects on ischemia/reperfusion injury in mice [16]. Several studies have revealed its important function on cardiovascular effects [17]. The present study aims to investigate the protective effect of Butorphanol tartrate on Hcy-induced stroke and BBB disruption, which will provide a novel treatment strategy for Hcy-induced stroke.

## Materials and methods

### Animals and grouping

Twelve 7-9-week male mice were obtained from Topgene Biological (Changsha, China) and adapted to the laboratory for 1 week. Animals were divided into 4 groups: the Control, Butorphanol tartrate, Hcy, and Hcy+ Butorphanol tartrate groups. In the control group, mice received vehicles throughout the study. In the Butorphanol tartrate group, animals received butorphanol tartrate intraperitoneally at a dosage of 20 μg/kg/day for 30 days. In the Hcy group, mice received Hcy subcutaneously at a dose of 0.03 μmol/g of body weight, twice a day at 8 h intervals for 30 days. In the Hcy+ Butorphanol tartrate group, animals received butorphanol tartrate and Hcy at doses mentioned above.

### Sodium fluorescein detection

Animals were injected with 0.1 mL 10% NaFI 45 min before being sacrificed, and then cardiac blood was collected. After transcardial perfusion with saline, the brain tissue and plasma were separated and frozen on dry ice followed by trichloroacetic acid (TCA) extraction. Subsequently, the absorbance at 480/520 nm was detected utilizing a microplate reader (Molecular Devices, California, USA), and the concentration of NaFI was calculated using the standard curve.

### Evan’s blue

Animals were intravenously injected with 4 mL/kg 2% Evan’s blue (MP Biomedicals, California, USA) dissolved in PBS. After 30 min circulation, mice were transcardially perfused with blank PBS until the flowing liquid from the right atrium was colorless. Then, brain tissues were collected and the Evans blue was fixed with formamide overnight at 50°C, followed by drying for 1 h at room temperature. The concentration of dye was quantified using the spectrophotometer (Molecular Devices, California, USA) at 611 nm.

### Real-time PCR

The total RNAs were extracted from tissues or cells using the TRIzol reagent (CW0580S, CWBIO, Beijing, China), followed by obtaining cDNA by transcribing 2 µg sample of RNA with a HiScript II Q RT SuperMix Kit (R223-01, Vazyme, Nanjing, China). The PCR reaction was performed utilizing the 7500 Real-Time PCR System (ABI, California, USA) with the SYBR Green PCR Master Mix (A4004M, Lifeint, Xiamen, China). The 2^−ΔΔCt^ method was used to determine the gene expression following normalization with GAPDH [18].

### Enzyme-linked immunosorbent assay (ELISA)

The production of IL-6 and TNF-α was detected using the commercial ELISA kits (MEIMIAN, Beijing, China). Brief, brain tissue homogenates and standards were implanted on 96-well plates. After incubation at 37°C for 90 min, plates were loaded with the conjugate solution to be incubated for another 1.5 h. Then, the TMB solution was added to be incubated for 15 min, followed by terminating the reaction using the stop solution. The absorbance at 450 nm was detected with a microplate reader (Molecular Devices, California, USA) [19].

### Immunostaining

Brain tissues were extracted from each animal and fixed using the 4% PFA for 24 hours, followed by being embedded with paraffin wax and cut into 5 μm tissue sections. Then, slides were deparaffined and hydrated. After being incubated with 3% H_2_O_2_ for 15 min, slides were blocked using the 5% bovine serum albumin (BSA) for half an hour, followed by adding the primary antibody against Claudin-5 (1:200, GeneTex, Beijing, China) for 1.5 h. Then, slides were washed and incubated with the horseradish peroxidase (HRP)-conjugated secondary antibody (1:500, GeneTex, Beijing, China) for 1 h, and images were captured using a light microscope (Keyence, Tokyo, Japan). The fluorescence intensity was assessed using the software Image J (NIH, USA).

### Cell culture, transduction, and treatment

Human brain vascular endothelial cells (HBVECs) were obtained from ATCC (Virginia, USA) and cultured in the DMEM medium containing 10% FBS and 10% growth factor, including epidermal growth factor, fibroblast growth factor-2, and heparin. To knock down KLF5 in HBVECs, shRNA targeting KLF5 was designed and synthesized by Genscript (Nanjing, China) and then packaged in lentiviral and transfected into HBVECs together with lip3000.

### Cell counting kit-8 (CCK-8) assay

HBVECs were implanted on a 96-well plate and incubated for 1 day, before adding 10 μL CCK8 reagents into each well. After being incubated for 2 h, the microplate reader (Molecular Devices, California, USA) was utilized to detect the absorbance at 450 nm to calculate the cell viability.

### Endothelial monolayer permeability

HBVECs were implanted on the upper chamber of the Transwell (Millipore, Massachusetts, USA), followed by adding 1 mg/mL FITC-dextran (MedChemExpress, New Jersey, USA) into the upper chamber of the Transwell. After incubation for 60 min, the microplate reader (Molecular Devices, California, USA) was utilized to obtain the absorbance of samples collected from the lower chamber at 492/520 nm to determine the permeability of the endothelial monolayer.

### Western blot assay

After extracting total proteins, quantification was performed on proteins, which were loaded onto the 12% SDS-PAGE. After separating for 1.5 h, proteins were transferred onto the PVDF membrane (Takara, Tokyo, Japan). After blocking with 5% skim milk, membranes were incubated with the primary antibody against Claudin-5 (1:1000, 20667-1-AP, Proteintech, Wuhan, China), KLF5 (1:1000, 18592-1-ap, Proteintech, Wuhan, China), and β-actin (1:1000, Proteintech, Wuhan, China), respectively. Subsequently, the membranes were incubated with the secondary antibody (1:2000, Proteintech, Wuhan, China) for 1.5 h, followed by being hatched with the ECL solution. Lastly, the expression level of target proteins was quantified with the Image J software [20].

### Statistical analysis

Mean ± SD was utilized to present data achieved from the present study, followed by analyzing data using the GraphPad software. Data among more than 3 groups were analyzed by the one-way Analysis of Variance (ANOVA) method. P < 0.05 was considered a significant difference.

## Results

In this study, we aimed to investigate the protective effects of butorphanol tartrate on Hcy-induced BBB disruption. In the *in vivo* model, butorphanol tartrate maintained BBB integrity rescued the expression of Claudin-5, and inhibited the expression of inflammation biomarkers. Importantly, the results in the *in vitro* model show that the protective effects of butorphanol tartrate on hcy-challenged HBVECs were mediated by KLF5.

### Effects of butorphanol tartrate on Hcy-induced increase in BBB permeability

**Figure 1.**
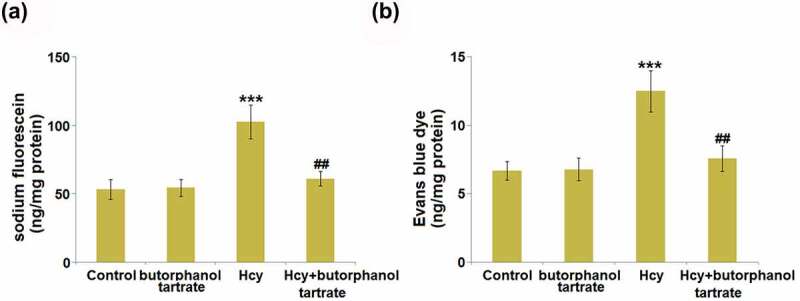
Effects of butorphanol tartrate on Hcy-induced increase in BBB permeability. Diffusion of (a) sodium fluorescein and (b) Evans blue dye (***, P < 0.001 vs. Control group; ##, P < 0.01 vs. Hcy group).

To evaluate the effects of butorphanol tartrate on BBB permeability, sodium fluorescein and Evans blue assays were conducted on each animal. The concentration of sodium fluorescein (Figure 1(a)) was slightly changed from 53.4 ng/mg protein in the control group to 54.5 ng/mg protein in the Butorphanol tartrate group, then further increased to 102.7 ng/mg protein in the Hcy group. After treating Hcy-dosed animals with Butorphanol tartrate, the concentration of sodium fluorescein was dramatically repressed to 61.2 ng/mg protein. Additionally, the concentration of Evans blue dye (Figure 1(b)) in the control, Butorphanol tartrate, Hcy, and Hcy+ Butorphanol tartrate groups was 6.7 ng/mg protein, 6.8 ng/mg protein, 12.5 ng/mg protein, and 7.6 ng/mg protein, respectively. These results suggest that the increased BBB permeability in Hcy-treated animals was significantly alleviated by butorphanol tartrate.

### Effects of butorphanol tartrate on the expression of claudin-5 in Hcy-treated mice brains

**Figure 2.**
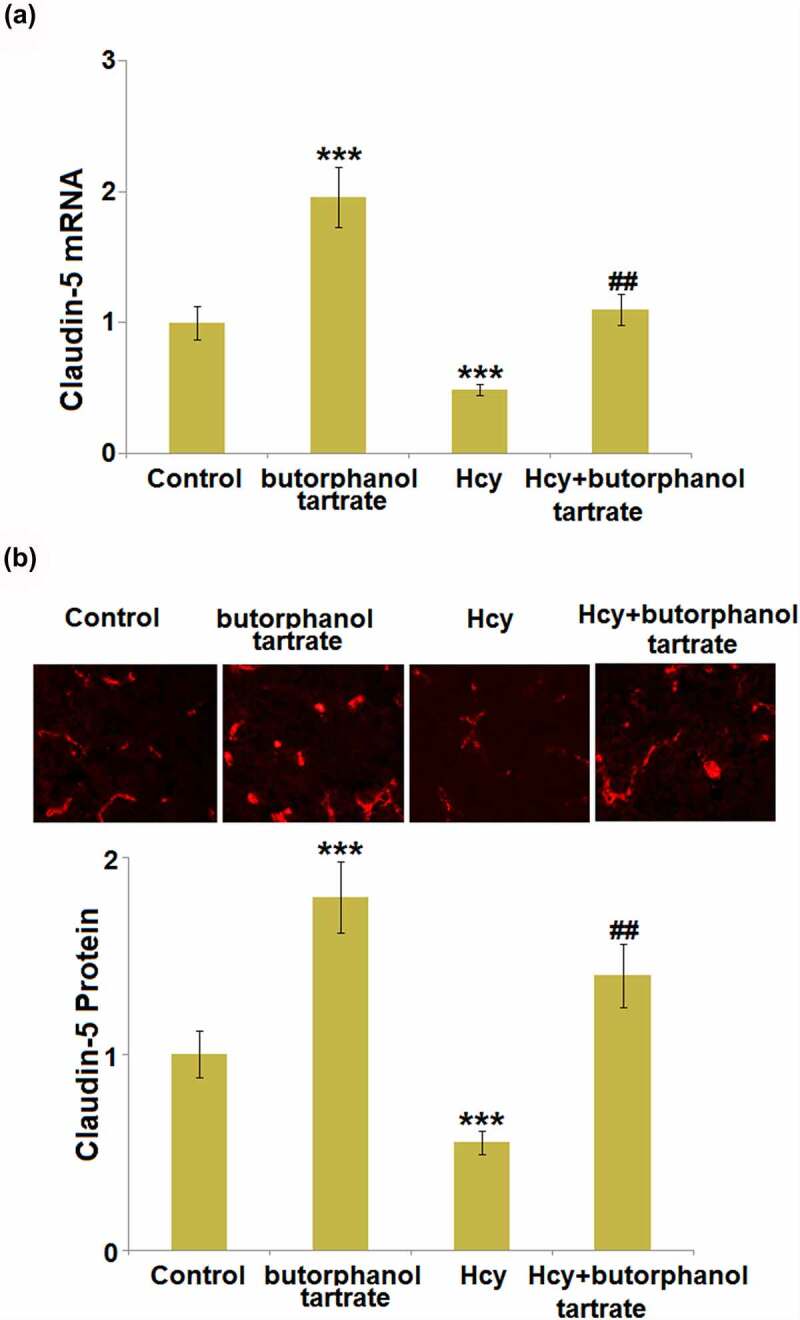
Effects of butorphanol tartrate on the expression of Claudin-5 in Hcy-treated mice brains. (a) mRNA level of Claudin-5 in the cortex; (b) Protein levels of Claudin-5 in the cortex were detected using immuno-fluorescent staining (***, P < 0.001 vs. Control group; ##, P < 0.01 vs. Hcy group).

Claudin-5 is an important tight junction protein located in the BBB and is critical for the maintenance of BBB integrity. We further measured the expression level of Claudin-5 in the brain tissue of each animal. Both at the mRNA and protein levels (Figure 2(a,b)), Claudin-5 was found significantly upregulated in normal mice after the treatment with butorphanol tartrate. Additionally, Claudin-5 was dramatically downregulated in Hcy-treated mice, which was greatly reversed by butorphanol tartrate, suggesting that the protective effect of butorphanol tartrate on BBB permeability might be associated with the upregulation of Claudin-5.

### Effects of butorphanol tartrate on the expression of inflammation biomarkers in Hcy-induced mice

**Figure 3.**
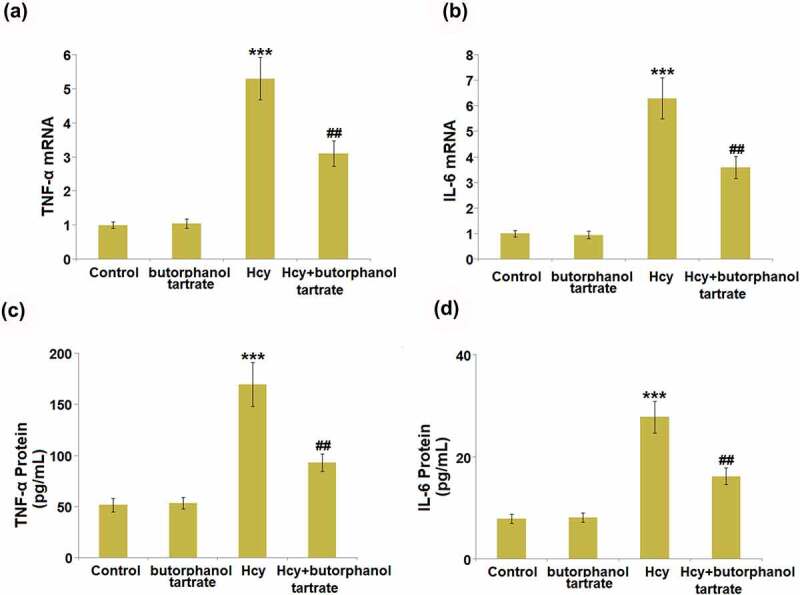
Effects of butorphanol tartrate on the expression of inflammation biomarkers in the brains of Hcy-induced mice. (a). mRNA levels of TNF-α; (b). mRNA levels of IL-6; (c) Protein levels TNF-α in the brain; (d) Protein levels of IL-6 (***, P < 0.001 vs. Control group; ##, P < 0.01 vs. Hcy group).

Severe inflammation is reported to be involved in Hcy-induced stroke. The concentrations of IL-6 and TNF-α in the brain of each animal were detected. In normal mice, the gene levels of IL-6 and TNF-α (Figure 3(a,b)) in the brain were not significantly changed by the administration of butorphanol tartrate. However, IL-6 and TNF-α were found greatly upregulated by Hcy, which was dramatically reversed by butorphanol tartrate. The protein level of TNF-α in normal mice (Figure 3(c)) was slightly changed from 51.6 pg/mL to 53.5 pg/mL by butorphanol tartrate, then greatly promoted to 169.8 pg/mL in Hcy-treated mice. Under the treatment of butorphanol tartrate, the concentration of TNF-α in Hcy-treated mice was dramatically reversed to 93.9 pg/mL. Additionally, the protein levels of IL-6 (Figure 3(d)) in the control, Butorphanol tartrate, Hcy, and Hcy+ Butorphanol tartrate groups were 7.9 pg/mL, 8.1 pg/mL, 27.8 pg/mL, and 16.2 pg/mL, respectively. These data collectively suggest that the severe inflammation in Hcy-treated mice was dramatically ameliorated by butorphanol tartrate.

### Cytotoxicity of butorphanol tartrate in HBVECs

**Figure 4.**
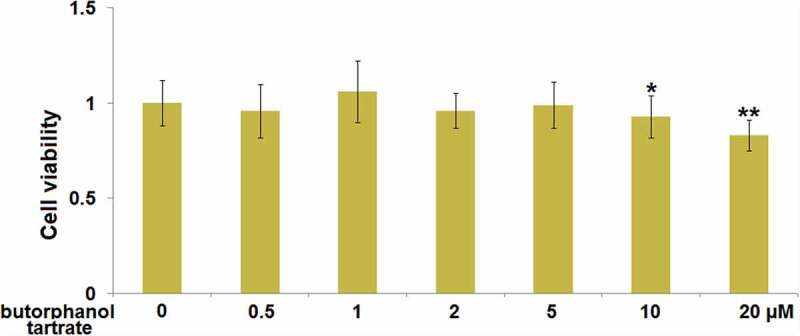
Cytotoxicity of butorphanol tartrate in HBVECs. Cells were treated with butorphanol tartrate (0.5 µM, 1 µM, 2 µM, 5 µM, 10 µM, 20 µM) for 24 hours. Cell viability was determined by CCK-8 assay (*, **, P < 0.05, 0.01 vs. Control group).

Brain vascular endothelial cells are the main components of the BBB [21]. We further investigated the potential therapeutic mechanism of butorphanol tartrate in HBVECs. Firstly, the optimized treatment concentration of butorphanol tartrate in HBVECs was investigated. HBVECs were treated with butorphanol tartrate (0.5 µM, 1 µM, 2 µM, 5 µM, 10 µM, 20 µM) for 24 hours, followed by evaluating the cell viability using the CCK-8 assay. We found that the highest concentration of butorphanol tartrate (Figure 4) that did not impact the cell viability of HBVECs was 5 µM. Therefore, in the subsequent experiments, 2 and 5 µM were chosen as the incubation concentrations of butorphanol tartrate in HBVECs.

### Effect of butorphanol tartrate on Hcy-induced brain endothelial monolayer permeability in HBVECs

**Figure 5.**
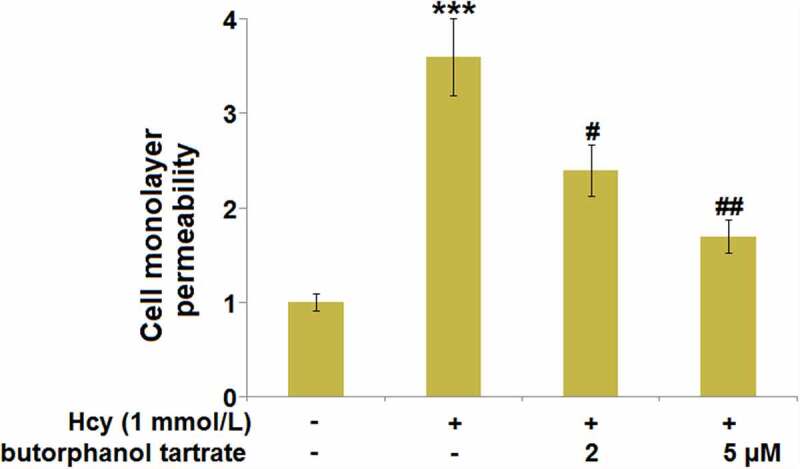
Effect of butorphanol tartrate on Hcy-induced brain endothelial monolayer permeability in HBVECs. Cells were treated with Hcy (1 mmol/L) in the presence or absence of butorphanol tartrate (2 µM, 5 µM) for 24 hours. Cell monolayer permeability was measured using a permeability assay (***, P < 0.001 vs. Control group; #, ##, P < 0.05, 0.01 vs. Hcy group).

To explore the effects of butorphanol tartrate on endothelial permeability, HBVECs were treated with Hcy (1 mmol/L) in the presence or absence of butorphanol tartrate (2 µM, 5 µM) for 24 hours, followed by evaluating the monolayer permeability using the FITC-dextran assay. We found that the monolayer permeability of HBVECs (Figure 5) was significantly promoted by Hcy, which was greatly declined by the treatment with 2 and 5 µM butorphanol tartrate, suggesting the increased endothelial permeability induced by Hcy was greatly alleviated by butorphanol tartrate.

### Effects of butorphanol tartrate on the expression of claudin-5 in Hcy-treated HBVECs

**Figure 6.**
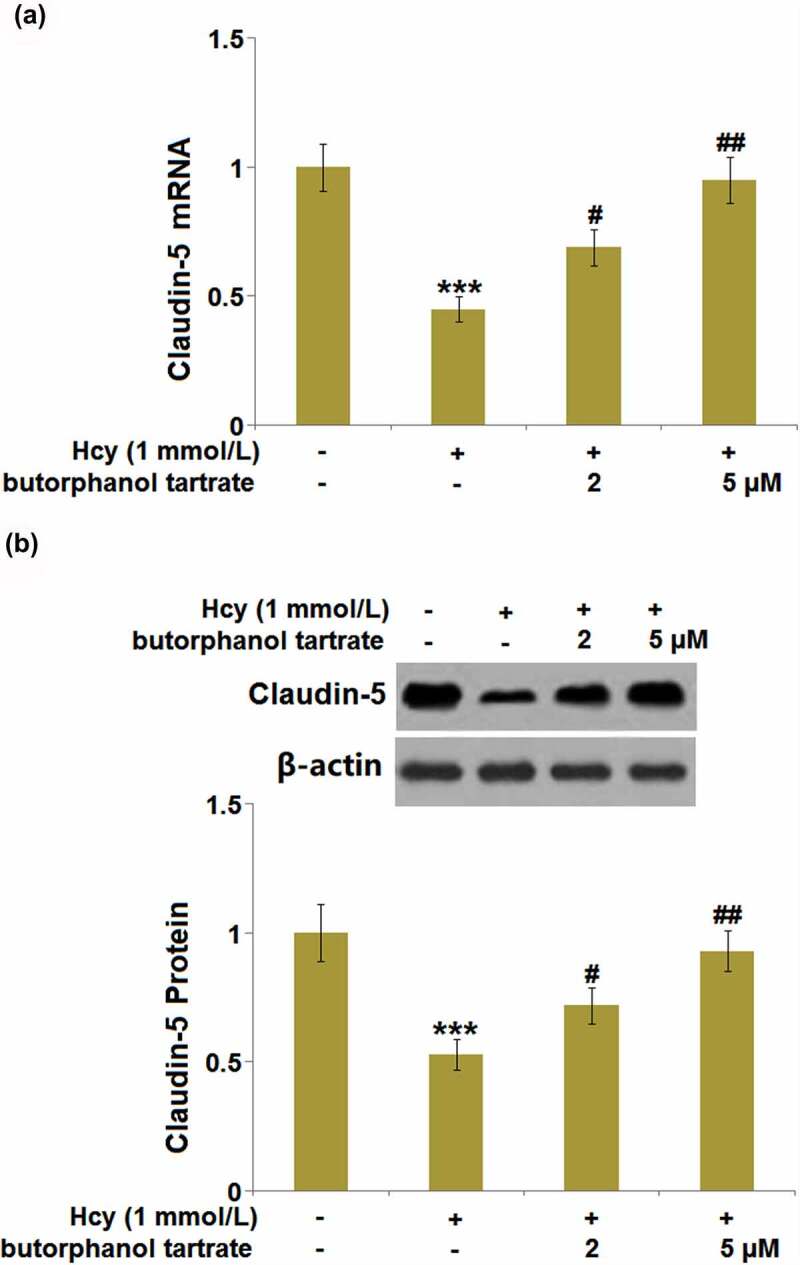
Effects of butorphanol tartrate on the expressions of Claudin-5 in Hcy-treated HBVECs. Cells were treated with Hcy (1 mmol/L) in the presence or absence of butorphanol tartrate (2 µM, 5 µM) for 24 hours. (a) mRNA Levels of Claudin-5 were determined; (b) Protein levels of Claudin-5 were determined (***, P < 0.001 vs. Control group; #, ##, P < 0.05, 0.01 vs. Hcy group).

As we have observed the impact of butorphanol tartrate on Claudin-5 in Hcy-treated animals, we further verified the regulatory effects of butorphanol tartrate on the expression level of Claudin-5 in Hcy-treated HBVECs. We found that the expression level of Claudin-5 (Figure 6(a,b)) in Hcy-treated HBVECs was dramatically repressed, then greatly rescued by 2 and 5 µM butorphanol tartrate, consistent with the observation in the animal experiments.

### Butorphanol tartrate rescued the reduction of KLF5 in Hcy-treated HBVECs

**Figure 7.**
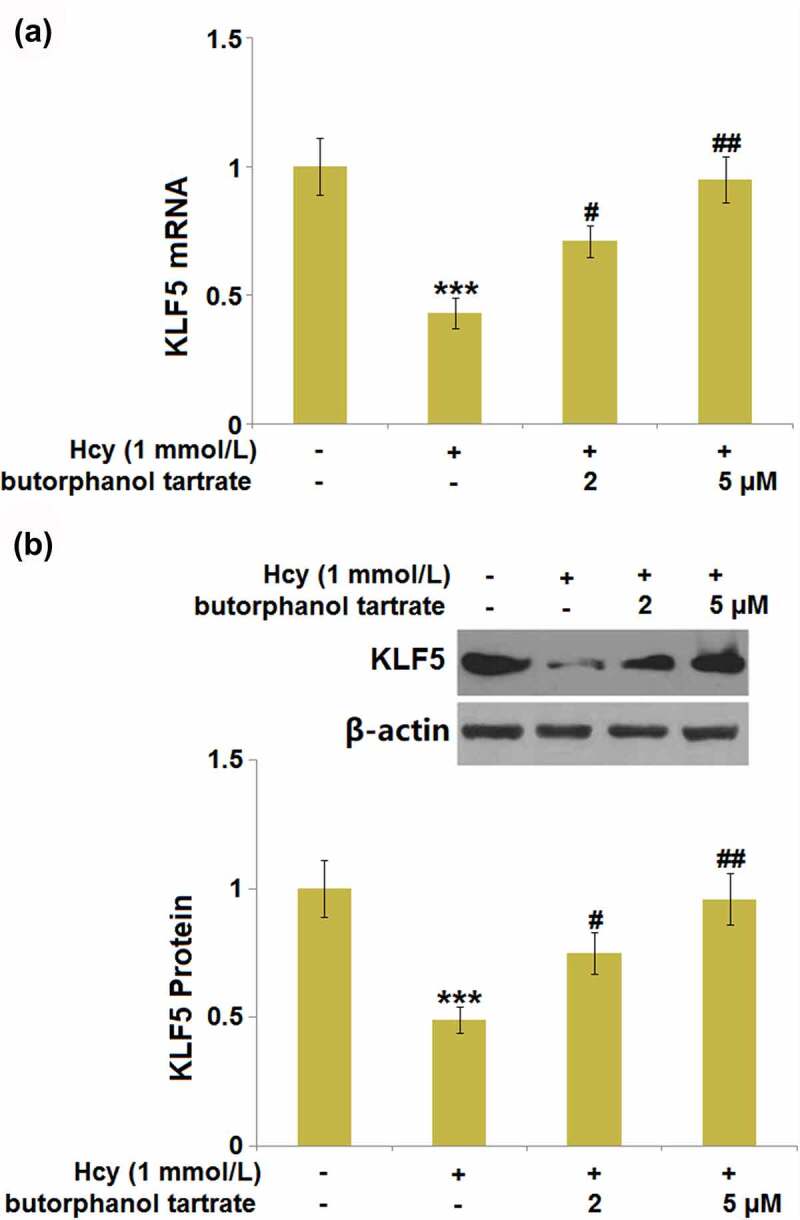
Butorphanol tartrate rescued the reduction of KLF5 in Hcy-treated HBVECs. Cells were treated with Hcy (1 mmol/L) in the presence or absence of butorphanol tartrate (2 µM, 5 µM) for 24 hours. (a). mRNA of KLF5; (b). Protein levels of KLF5 (***, P < 0.001 vs. Control group; #, ##, P < 0.05, 0.01 vs. Hcy group).

KLF5 is an important transcriptional factor that maintains BBB and tight junction integrity ^21^. We further explored whether butorphanol tartrate showed regulatory effects on the function of KLF5. Following stimulation with Hcy, KLF5 was dramatically downregulated (Figure 7(a,b)) in HBVECs, then obviously rescued by 2 and 5 µM butorphanol tartrate, suggesting a promising regulatory effect of butorphanol tartrate on the transcriptional factor KLF5.

### Knockdown of KLF5 rescued the effect of butorphanol tartrate in Hcy-stimulated HBVECs

**Figure 8.**
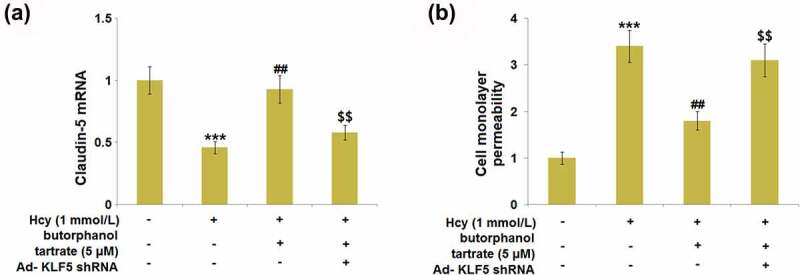
Knockdown of KLF5 abolished the protective effects of Butorphanol tartrate in the expression of Claudin-5 and endothelial permeability. Cells were transduced with Ad-KLF5 shRNA, followed by stimulation by Hcy (1 mmol/L) in the presence or absence of butorphanol tartrate (5 µM) for 24 hours. (a). mRNA expression of Claudin-5; (b). Cell monolayer permeability (***, P < 0.001 vs. Control group; ##, P < 0.01 vs. Hcy group; $$, P < 0.01 vs. Hcy+butorphanol tartrate group).

Cells were transfected with Ad- KLF5 shRNA, followed by stimulation with Hcy (1 mmol/L) in the presence or absence of butorphanol tartrate (5 µM) for 24 hours. We found that the repressed expression level of Claudin-5 (Figure 8(a)) in the Hcy-treated HBVECs was greatly promoted by butorphanol tartrate, which was dramatically abolished by the knockdown of KLF5. Additionally, the increased endothelial monolayer permeability (Figure 8(b)) in Hcy-treated HBVECs was dramatically declined by butorphanol tartrate, then rescued by knocking KLF5 down. These data collectively suggest that the function of butorphanol tartrate in Hcy-stimulated HBVECs was rescued by the downregulation of KLF5.

## Discussion

Under a normal physiological state, biomacromolecules, such as fibrinogen, fibrin, immunoglobulin G (IgG), and low molecular dextran, are blocked by the BBB from entering brain tissue. However, once the BBB is disrupted, its permeability is increased, which is mainly induced by injury to endothelial cells, disruption of tight junctions, enhancement of vesicles transshipments, and the swelling of astrocytes [22,23]. It is reported that BBB disruption results in the invasion of exogenous contrast medium, horseradish peroxidase (HRP), and Evans blue into the brain tissue [24]. We found that the diffusions of sodium fluorescein and Evans blue were significantly aggravated in Hcy-treated mice, indicating a destructive effect of Hcy on BBB permeability, which was consistent with observations claimed in previous researches [25,26]. The increased production of inflammatory factors observed in the brain tissues of Hcy-treated mice further confirmed the disrupted BBB. Under the 30-day consecutive treatment of 20 μg/kg/day butorphanol tartrate, the increased BBB permeability in Hcy-treated animals was alleviated, accompanied by the declined secretion of inflammatory factors in the brain. *In vitro* assays further verified that the increased endothelial permeability in HBVECs induced by Hcy was greatly reversed by butorphanol tartrate. These observations suggest that butorphanol tartrate might be a potential therapeutic agent for the treatment of BBB disruption-associated stroke. In addition, no significant impact of butorphanol tartrate was observed on BBB permeability in normal mice, implying that the safety of butorphanol tartrate for the treatment of stroke is guaranteed.

Tight junctions are composed of a series of related proteins, such as occludins, claudins, junctional adhesion molecule-A (JAM-A), Zonula occludens-1 (ZO-1), and host proteins that connect the cytoskeleton [27]. Claudins are a group of proteins transcribed by multiple genes, including Claudin-3, Claudin-5, and Claudin-12 located on the BBB [28,29]. Claudins comprise an important part of the BBB by interacting with the adhesion molecules at the sites of tight junctions [30]. Previous research indicated that death was found in Claudin-5 knockout mice and blockage of the BBB to molecules smaller than 800 Da was relieved [28], indicating that Claudin-5 plays a critical role in the maintenance of BBB integrity. In the brain injury model induced by ischemia-reperfusion, Claudin 5 and Occludins are degraded by matrix metalloproteinases (MMPs) and accumulate in astrocytes and neurons around blood vessels through phagocytosis [31,32]. We found that in both Hcy-treated mice and HBVECs, downregulated Claudin-5 was observed. This regulatory effect of Hcy on Claudin-5 was previously reported by Pradip [12]. Our data further confirmed that the function of butorphanol tartrate on BBB and endothelial permeability was accompanied by the upregulation of Claudin-5. We found that KLF5 was downregulated in Hcy-treated HBVECs, which was reversed by butorphanol tartrate. Further investigation suggested that the function of butorphanol tartrate in Hcy-stimulated HBVECs was reversed by the knockdown of KLF5, implying that the therapeutic effect of butorphanol tartrate is dependent on KLF5. The mechanism of inflammation changes caused by KLF5 is complicated. Generally, it is considered that KLF5 plays dual roles in regulating the inflammatory response. On one hand, KLF5 could increase the expressions of pro-inflammatory cytokines such as TNF-α, IL-1β, and IL-6 at both protein and mRNA levels, by activating the NF-κB pathway [33–35]. However, an anti-inflammatory role of KLF5 is also reported. Using an *in vitro* myocardial oxygen-glucose deprivation/reperfusion (OGD/R) injury model, Li et al found that KLF5 suppressed the expression of several pro-inflammatory mediators via the PPAR-γ/PGC-1α/TNF-α signaling pathway [36]. Moreover, a recent study demonstrated that overexpression of KLF5 could inhibit puromycin aminonucleoside (PAN)-induced apoptosis of podocytes via downregulating the p38 MAPK signaling pathway, which is also an important modulator in various inflammatory diseases [37]. Therefore, further studies are needed to clarify the underlying mechanisms of KLF5 in regulating inflammatory responses in various diseases. In future work, the direct interaction between butorphanol tartrate and KLF5 will be researched to explore the molecular mechanism underlying the therapeutic property of butorphanol tartrate on the BBB.

## Conclusion

Collectively, our results demonstrate that butorphanol tartrate displays beneficial capacities on Hcy-induced BBB disruption by upregulating the expressions of KLF5 and Claudin-5, suggesting a novel pharmacological function of butorphanol tartrate on brain cardiovascular diseases such as stroke.

## Data Availability

The data that support the findings of this study are available from the corresponding author upon reasonable request.
